# Progress in Ophthalmology

**Published:** 1902-11-22

**Authors:** 


### Progress in Ophthalmology.

(*Concluded from page 114.*)

**Detachment of the Retina.**—At the July meeting of the Ophthalomological Society Mr. Higgins[5] read notes of a case of detachment of the retina, which had become cured. Patient was aged 36, had been under his care for ten years for high myopia, and had three years ago practically lost the sight of her left eye from detachment of the retina. One year before the communication she had first noticed obscuration of the field of her right eye. There was a doubtful history of injury some weeks before. Detachment of the retina at its lower part was discovered. She was treated for nearly a month in hospital, lying in the prone position, with mercurial inunction and vapour baths, and she left with some improvement. Two weeks later her sight returned; while lying down in the afternoon she went to sleep for some hours, and on waking found the sight as good as it had been before the detachment occurred. Ophthalmoscopic examination showed some floating shreds in the vitreous, but no sign of detachment. Nearly nine months since the detachment appeared, and eight months since the recovery of vision, she could see with correction $\frac{6}{24}$ and Jj. The field was almost normal, and there was no sign of the detachment. He doubted very much whether the treatment adopted in his case had anything to do with the recovery of vision.

**Cataract Extraction in India.**—Captain Elliott, I.M.S.,[2] in a paper on an analysis of a series of cataract extractions in natives, points to the many disadvantages of extraction without iridectomy, and considers that the combined method, viz., extraction with iridectomy is preferable and gives better results. He also draws attention to this point, which is worth noting: the lower visual results in the cases of the combined method of extraction are easily explained, when we remember that in this series, iridectomies were done at the time of the operation, because the cases were complicated ones. The simple operations thus represent a more favourable class of cases than do the others. In favour of the simple operation it is usually advanced:—first, that it gives better visual results than the combined operation does; second, that it provides a more active pupil; third, that the dazzling caused by the coloboma is avoided; fourth, that there is less mutilation of the eye. With the first contention he does not agree; as to the second, he finds that the pupil in a well-performed case of the combined operation is very active, and he doubts if there is anything in favour of this proposition. As to the third, the upper lid covers the coloboma, and the patients do not seem to be aware of its presence. As to the fourth, it appears to him to appeal to a sentiment which is not founded on sound surgical principles. He believes that the after course of the combined operation cases is more even and uneventful than is that of the simple operation. Against the simple operation there is always the grave danger of prolapse of the iris. Even Knapp admits to the occurrence of prolapse in 7·6 per cent. of his latest cases; most operators admit more, and he believes that the 14 per cent. met with by him in his series is not excessive. A wider experience than the cases now under review affords has convinced him that the danger of losing eyes after prolapse of the iris is a very real one, and the anxiety for the three or four days after operation is too dear a price to pay, except for solid advantages which he fails to see the simple operation can afford. Neither eserine or atropin avail to prevent the accident. Iridectomy must now frequently be performed under very disadvantageous circumstances; if an anæsthetic be given the eyeball rotates upwards and the effort to pull it down by means of the forceps threatens the integrity of the hyaloid and with it the safety of the eye; again, if we rely on cocaine alone, the strangled iris is so hyperæsthetic that the patient finds it hard to keep quiet and his sudden spasmodic movements make the operation one of the gravest anxiety. With regard to the resulting vision he gives an explanation of the rough and ready method which is almost necessary in these native cases. "The vision of the patients was tested after operation by ascertaining at what distance they could count miniature bull's-eyes. These consist of black dots on a white ground, which are shown to the subject through an aperture in a piece of cardboard. The examiner can thus show as many or as few as he wishes. He finds by experiment that the dots can be counted by a normal eye at 10 metres. Patients were tested up to six metres and not beyond. The test is that in use at the Madras Eye Hospital. It is a convenient one when dealing with patients whose intelligence and education are both of a very low order. Several patients absconded rather than submit to being tested; several left without warning as soon as they found they were in possession of good sight. The native women especially object to wearing glasses." This explanation would answer an objection we made not long ago in these articles to the scant amount of visual results which were given in these Indian statistics.

[5] Brit. Med. Jour., July 19, 1902.

